# Phenotyping the physiological and biochemical changes of nopal cladodes during early postharvest storage

**DOI:** 10.3389/fpls.2026.1648343

**Published:** 2026-02-10

**Authors:** Jesús Rodolfo Valenzuela-García, Alejandro Isabel Luna-Maldonado, Mario Alberto Méndez-Dorado, Martín Cadena-Zapata, Juan Arredondo-Valdez, Mónica Gabriela Valenzuela Carrizales, Juan Antonio López López, Blanca Elizabeth de la Peña-Casas, Gilbert Fresh López-López, Alain Buendía-García, Juan Florencio Gómez-Leyva

**Affiliations:** 1 Universidad Autónoma de Nuevo León, Facultad de Agronomía, Departamento de Ingeniería Agrícola y de los Alimentos, General Escobedo, Nuevo León, Mexico; 2 Universidad Autónoma Agraria Antonio Narro, Departamento de Maquinaria Agrícola, Saltillo, Coahuila, Mexico; 3 Instituto Tecnológico de Saltillo, Departamento de Mecatrónica, Saltillo, Coahuila, Mexico; 4 Universidad Autónoma Agraria Antonio Narro. Departamento de Suelos Periférico Raúl López Sánchez, Torreón, Coahuila, Mexico; 5 Laboratorio de Biología Molecular, TecNM—Instituto Tecnológico de Tlajomulco (ITTJ), Tlajomulco de Zúñiga, Jalisco, Mexico

**Keywords:** antioxidant activity, cladode biochemical composition, *Opuntia ficus-indica*, phenotypic characterization, postharvest physiology

## Abstract

This study assessed short-term postharvest changes in morphological, colorimetric, and biochemical traits of *Opuntia ficus-indica* (nopal) cladodes across four commercial categories (Cambray, C, B, and A) over 10 days, with measurements at 3, 6, and 9 days. Parameters included weight, size, texture, thickness (base, center, tip), color (hue angle, chroma), chlorophyll a, b, total, pheophytins, antioxidant capacity (ABTS, DPPH, FRAP), and total phenolic and flavonoid contents. ANOVA and Tukey’s HSD tests were applied. Size, chroma, and hue angle did not vary significantly across categories. Thickness remained stable for categories C and B; Cambray varied at base, center, and tip; A varied at base and tip. Weight changed in Cambray and A but remained constant in C and B. Firmness differences were observed in Cambray and B, but not in C and A. Category B had the highest chlorophyll a (412 mg/100 g DW), chlorophyll b (327 mg/100 g DW), total chlorophyll (739 mg/100 g DW), and pheophytins (9229.2 mg/100 g DW). Ascorbic acid was highest in A (7.81 mg/g). ABTS values were highest in C (3.51 mmol TE/100 g DW) and A (3.43 mmol TE/100 g DW), while DPPH (0.63 mmol TE/100 g DW) and FRAP (1.70 mmol TE/100 g DW) peaked in C. Polyphenols were highest in Cambray (103.8 GAE/100 g DW) and C (99.2 GAE/100 g DW), and flavonoids were highest in A (280.5 mg QE/100 g DW). The optimal category showed stable size, firmness, thickness, and color, alongside the highest chlorophyll and pheophytin contents. These findings reveal postharvest dynamics and biochemical diversity in nopal, providing valuable insights for agricultural management and industrial applications.

## 1 Introduction

The global population is projected to surpass 9 billion by 2050 (UN, 2023), highlighting the urgent need to diversify food systems and explore sustainable, climate-resilient crops. In this context, *Opuntia ficus-indica* (OFI), commonly known as prickly pear cactus, has gained attention for its adaptability to arid and semi-arid environments, low water requirements, and high nutritional value (Naorem et al., 2024). In Mexico, OFI is widely known as *nopal* and holds cultural, economic, and nutritional significance (Das et al., 2021; González-Stuart and Rivera, 2019). The term *nopal* typically refers to the young, tender cladodes consumed as vegetables, whereas the plant as a whole is also recognized by names such as prickly pear, spineless cactus, fodder cactus, *Indian opuntia*, green gold, and fig opuntia (Bakewell-Stone, 2022). OFI is considered the most economically important cactus in global agriculture, valued not only for its edible fruits but also for its cladodes, which serve as food for humans and fodder for livestock, depending on their stage of maturity (Kiesling, 1998; Casas and Barbera, 2002). Cladodes, the photosynthetic stem segments of the cactus, are rich in fiber, vitamins, minerals, phenolic compounds, and antioxidants. Their composition varies according to species, age, environmental conditions, and postharvest handling (Elshehy et al., 2020; Hernández-Urbiola et al., 2010). Several studies have reported the presence of essential amino acids, high levels of vitamin C, phenolics, flavonoids, and antioxidant activity in OFI cladodes, supporting their use in food, nutraceutical, and pharmaceutical products (Barba et al., 2022; de Wit and Fouché, 2021; Missaoui et al., 2020; Martins et al., 2023; Silva et al., 2021; Oluwole et al., 2022).

**Figure 1:**
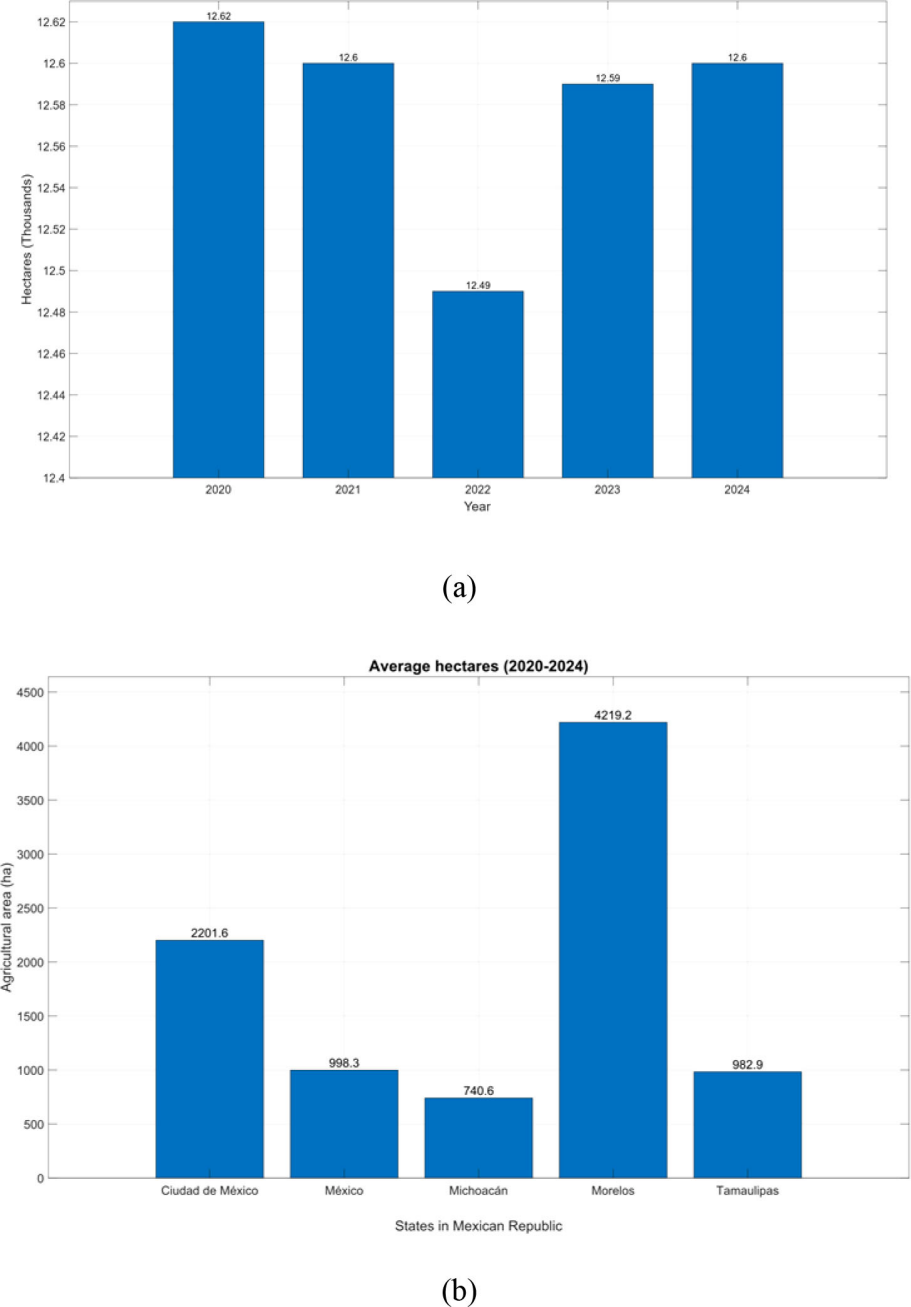
**(A)** Number of hectares planted with nopal in Mexico. **(B)** Main nopal-producing states in Mexico (SIAP, 2020; 2021; 2022; 2023; 2024).

Mexico leads global nopal production, with an average of 12,577 hectares cultivated annually between 2020 and 2024, yielding approximately 877,525 tons per year. The main producing states are Morelos, Mexico City, State of Mexico, Tamaulipas, and Michoacán, which together account for 72% of the national cultivated area (SIAP, 2020; 2021; 2022; 2023; 2024) (**Figure 1**). Although various studies have analyzed the nutritional and chemical composition of *Opuntia* species, fewer have addressed how these parameters change shortly after harvest. In commercial contexts, understanding the short-term physiological changes that occur during the early postharvest period is essential for improving product quality, extending shelf life, and informing best handling practices.

This study aimed to evaluate short-term postharvest changes in *Opuntia ficus-indica* (Atlixco variety) cladodes across four commercial categories (Cambray, C, B, and A) over a 10-day period, during which 95% of the harvested cladodes are typically marketed. The analysis included morphological traits (weight, length, thickness, and texture), colorimetric parameters (hue angle and chroma), which are observable and valued by consumers at the time of purchase, and biochemical composition (chlorophyll a and b, pheophytins, phenolic and flavonoid content, and antioxidant capacity).This is because these compounds are responsible for flavor and aroma; moreover, they play important roles in the human body, such as reducing free radicals, and exhibit anti-inflammatory, antimicrobial, and anticancer activities, thereby helping to reduce the risk of developing chronic diseases. By examining variations over time and category, this research provides insights into the physiological behavior of nopal cladodes during early postharvest storage, with potential applications in quality control and value-added processing.

Therefore, the objective of this study was to analyze changes in the physical properties of different cladode categories over the evaluated time period and to determine differences in chemical properties among cladode sizes.

## 2 Materials and methods

### 2.1 Plant material

Cladodes of the Atlixco variety were collected from four commercial categories of nopal (*Opuntia ficus-indica*): Cambray, C, B, and A. Each category included 30 individual cladodes, totaling 120 samples. Harvesting was conducted manually between 9:00 a.m. and 11:00 a.m. on September 24, 2024, from a plantation located in Ejido El Pilar, in the municipality of General Cepeda, Coahuila, Mexico (25°23’0.69” N, 101°27’19.96” W). After harvesting, samples were individually stored in paper bags and maintained at ambient temperature (23–26°C) prior to analysis. Cladodes classification followed the specifications outlined in the Mexican Official Standard NMX-FF-068-SCFI-2006: Cambray (7.0–11.0 cm), Category C (11.1–18.0 cm), Category B (18.1–25.0 cm), and Category A (≥25.1 cm), based on cladode length. The following parameters were evaluated: firmness, weight, external color, and thickness (at the base, center, and tip); chlorophyll content (a, b, and total); ascorbic acid concentration; and titratable acidity. Firmness, weight, color, and thickness measurements were conducted at the Laboratory of the Faculty of Agronomy, Autonomous University of Nuevo León. Chlorophyll content, ascorbic acid, and titratable acidity were analyzed at the Molecular Biology Laboratory of the Technological Institute of Tlajomulco.

### 2.2 Experimental design

The laboratory tests were conducted on three dates: Time 1 (27/09/2024), Time 2 (30/09/2024), and Time 3 (03/10/2024), with three-day intervals between measurements. Cladodes from all categories were harvested on the same day, and the following assessments were performed: morphological, colorimetric, and biochemical analyses.

### 2.3 Morphological measurements

All fresh cladodes from each of the four categories (Cambray, C, B, and A) were harvested on September 27, 2024, to evaluate morphological, colorimetric, and biochemical parameters.

### 2.4 Cladode size

Cladode length was used as a proxy for size and was measured using a standard ruler from the base to the tip of each sample at all three time points.

### 2.5 Cladode thickness

Thickness was measured at three points on each cladode: the base, center, and tip. A precision digital caliper with a sensitivity of 0.01 mm was used to ensure accuracy.

### 2.6 Cladode weight

Cladode weight was measured in grams using a digital balance (Adam Core Compact Grain Balances CQT 1752GR), with a 2 kg capacity.

### 2.7 Cladode firmness

Firmness was assessed at specific points along the central longitudinal section of the cladodes using a texture analyzer (TA.XT Plus, Stable Micro Systems Ltd., Godalming, UK) equipped with a 5 mm cylindrical probe (P/3). Puncture resistance was evaluated for cladodes of each size category. Test parameters were as follows: test speed of 20 mm/s and a penetration distance of 10 mm.

### 2.8 Color

Color measurements were performed using the CHIN SPEC color meter, which has an aperture of 1.25 cm. The L*, a*, and b* values were measured from the middle section of one side of each cladode evaluated. The L* value indicates the lightness of the color tone, measured on a scale from 0 (black) to 100 (white). The hue angle or angle tone ( ${\text{tan}}^{-1\;}{\text{b}}^{*}\;/{\text{a}}^{\text{* }}$) and chroma or color saturation ( $\sqrt{{\left({\text{a}}^{*}\right)}^{2}+{\left({\text{b}}^{*}\right)}^{2}}$) were determined. Chroma represents the intensity or vividness of the color. The L* value is directly interpreted as luminosity or brightness.

### 2.9 Content of photosynthetic pigments

The content of photosynthetic pigments was evaluated by the spectrophotometric method described by Hynstova et al. (2018). The pigment of 0.5 g of fresh tissue obtained from the central cladode of each experimental unit with a scalpel were extracted with 3 mL of 85% acetone. The samples were refrigerated for 24 hours, then macerated and filtered through cheesecloth. The resulting extract was diluted to a final volume of 10 mL with 85% acetone. Absorbance was measured at 470, 645 and 662 nm, and chlorophyll a, chlorophyll b, and total chlorophyll concentrations were calculated using standard equations. Results were expressed in mg 100 g^-1^ fresh weight.

### 2.10 Ascorbic acid

Ascorbic acid was measured using the volumetric method. For this, 20 g of sample were used, and 10% hydrochloric acid was added while processing the tissue in a mortar. Then, 100 mL of distilled water was added. The sample was filtered and titrated with Thielman’s Reagent until a pink color appeared (three replicates per sample). The final volume after filtering was determined by summing the three aliquots used in the titration. The ascorbic acid content was then calculated and expressed as mg 100 g^-1^ of sample.

### 2.11 Titratable acidity

Titratable acidity was determined using a standard volumetric method. For each sample, 50 g of cladode tissue was chopped into small squares and placed in a 250 mL beaker. Then, 100 mL of distilled water was added, and the mixture was left to stand at room temperature for 24 hours. After extraction, the mixture was filtered, and a 10 mL aliquot of the filtrate was transferred into a 125 mL Erlenmeyer flask. Three to five drops of phenolphthalein indicator were added to each aliquot, and the solution was titrated with 0.1 N NaOH until a persistent deep pink endpoint was reached. Each sample was analyzed in triplicate. Titratable acidity was calculated and expressed as a percentage of citric acid equivalents (% citric acid).

### 2.12 Extract preparation

200 mg of freeze-dried cladode powder was placed in a 2 mL microcentrifuge tube. Then, 1.8 mL of extraction solution (water: ethanol, 30:70 v/v) was added. The mixture was vortexed for 20 s, sonicated in an ultrasonic bath (Branson M1800H-E, USA) at 40 kHz, 220 V, and 30°C for 10 min, and centrifuged at 12,000 rpm for 10 min at 4°C. The resulting supernatant was collected for further analysis.

### 2.13 Total phenolic content

The total phenolic content of cladodes was determined using the Folin–Ciocalteu method, as described by Singleton et al. (1999). Briefly, 200 µL of distilled water was mixed with 250 µL of Folin–Ciocalteu solution (1 N) and 50 µL of cladode extract or with gallic acid (20–180 µg mL^−1^) as a standard. After incubation for 5 min at room temperature, 500 µL of sodium carbonate solution (15% Na_2_CO_3_) was added. The mixture was thoroughly mixed and incubated in the dark at 45°C for 15 min. Subsequently, 200 µL of each reaction mixture was measured at 760 nm using a BioTek Synergy HTX microplate reader (Winooski, VT, USA). Results were expressed as milligrams of gallic acid equivalent per gram (mg GAE g^−1^) of dry weight and are reported as mean ± standard deviation of three independent experiments performed in triplicate.

### 2.14 Determination of total flavonoid content

The total flavonoid content was determined using a colorimetric method described by Dewanto et al. (2002). Briefly, 0.25 mL of spinach extract or (+)-catechin standard solution was mixed with 1.25 mL of distilled water in a test tube, followed by the addition of 75 μL of 5% NaNO_2_ solution. After 6 minutes, 150 μL of 10% AlCl_3_·6H_2_O solution was added, and the mixture was allowed to stand for an additional 5 minutes before adding 0.5 mL of 1 M NaOH. The final volume was adjusted to 2.5 mL with distilled water, and the mixture was thoroughly mixed. Absorbance was measured immediately at 510 nm against a blank using a Lambda XLS spectrophotometer (PerkinElmer) and compared with similarly prepared quercetin standards of known concentrations. The assay was performed according to the Dowd method, as modified by Arvouet-Grand et al. (1994). A standard calibration curve was prepared using quercetin (20–100 mg), and results were expressed as milligrams of quercetin equivalents in milligrams per 100 grams (mg QE/100 g) of dry extract.

### 2.15 Antioxidant capacity

Antioxidant capacity was evaluated using ABTS, DPPH, and FRAP assays. Results are expressed as millimoles of Trolox equivalents per 100 g of dry weight (mmol TE/100 g DW).

#### 2.15.1 DPPH• Method (2,2-diphenyl-1-picrylhydrazyl)

Antioxidant activity was determined following the protocol of Fukumoto and Mazza (2000). A 150 μM DPPH• solution was prepared in 80% methanol. The assay was performed in 96-well microplates (ICN Biomedicals Inc.) by adding 22 μL of sample and 200 μL of DPPH• solution per well. Each concentration (0–500 μM) was tested in triplicate, using at least seven different concentrations. Microplates were kept covered and protected from light at room temperature (~22°C), and absorbance was measured at 30, 180, and 360 minutes using an MRX microplate reader at 520 nm. For caffeic acid, the incubation was extended to 48 hours to ensure complete reaction. Antiradical activity was calculated from the initial slope of the linear regression curve (r² > 0.800) and expressed as μmol Trolox equivalents per gram of dry weight (μmol TE/g DW). Results are presented as mean ± standard deviation of eight replicates.

#### 2.15.2 ABTS•+ Method (2,2’-azino-bis(3-ethylbenzothiazoline-6-sulfonic acid))

The ABTS assay was adapted for dried spinach biomass. The ABTS•+ radical was generated and diluted in ethanol to an absorbance of 0.700 at 734 nm before mixing with the samples. A total of 20 μL of extract and 200 μL of ABTS•+ solution was added to each well of a 96-well microplate, and absorbance was measured after 6 minutes. Results are expressed as mean ± standard deviation of eight replicates, in μmol Trolox equivalents per gram of dry weight (μmol TE/g DW).

#### 2.15.3 FRAP method (Ferric Reducing Antioxidant Power)

The FRAP reagent was prepared by combining acetate buffer (pH 3.6), TPTZ (2,4,6-tripyridyl-s-triazine), and ferrous sulfate (Fe_2_SO_4_). Dried biomass samples were extracted and added to the FRAP reagent, followed by incubation at 37°C for 30 minutes. Absorbance was measured at 593 nm, and results are reported as mean ± standard deviation of eight replicates, in μmol Fe^2+^ equivalents per gram of dry weight (μmol eq. Fe^2+^/g DW).

For antioxidant capacity, standard curves were created with gallic acid as a reference and are therefore reported as gallic acid equivalents. The equations and correlation coefficients for each method are reported below: ABTS: y = -0.0179x + 0.5588, r^2^ = 0.987, DPPH: y = -0.012x + 1.021, r^2^ = 0.994, FRAP: y = 0.034x - 0.0302, r^2^ = 0.998. Each analysis was performed in triplicate.

### 2.16 Statistical analysis

Analysis of variance (ANOVA) was used to assess statistically significant differences in cladode weight, size, texture, and thickness across different time points within each category. Additionally, ANOVA was also used to evaluate variations in colorimetric and biochemical parameters. When a significant effect of time was detected, Tukey’s Honestly Significant Difference (HSD) *post-hoc* test was applied to pinpoint which time points differed significantly. All statistical analyses were conducted using Minitab 20.3.

## 3 Results

### 3.1 Cladode size

The mean size of cladodes for each category (Cambray, C, B, and A) at each time point is shown in **Table 1**.

**Table 1:** Cladode size (cm) in September 27, September 30, and October 3, 2024.

Category	Time 1 Mean (SD)	Time 2 Mean (SD)	Time 3 Mean (SD)
Cambray	10.17 (1.56) a	10.28 (1.62) a	11.07 (0.69) a
C	16.65 (2.86) a	16.17 (2.54) a	15.84 (1.31) a
B	22.65 (1.25) a	22.50 (1.29) a	23.28 (1.96) a
A	28.12 (1.59) a	27.96 (1.38) a	29.49 (2.67) a

SD, Standard deviation. Values are expressed as mean ± standard deviation (SD). Means followed by the same lowercase letter within a column are not significantly different, while means followed by different lowercase letters indicate significant differences among categories at p < 0.05.

ANOVA results indicated a significant effect of time on cladode size for the Cambray category (F = 3.29, df = 2, p = 0.029). Tukey’s HSD *post hoc* tests showed that the size of Cambray cladodes was significantly greater at Time 3 (11.07 cm) compared to Time 1 (10.17 cm) and Time 2 (10.28 cm). No significant effect of time on cladode size was observed for categories C (*p* = 0.736), B (*p* = 0.494), and A (*p* = 0.184).

### 3.2 Cladode thickness

The data for cladode thickness at the base, center, and tip are presented in **Table 2**.

**Table 2:** Cladode thickness (mm) in September 27, September 30, and October 3, 2024.

Category	Location	Time 1 Mean (SD)	Time 2 Mean (SD)	Time 3 Mean (SD)
Cambray	Base	16.62 (1.31) a	15.09 (1.49) b	16.78 (1.20) a
	Centre	11.27 (1.63) a	9.38 (1.18) b	9.59 (1.21) b
	Tip	8.16 (0.82) a	7.45 (0.63) ab	7.07 (0.95) b
C	Base	19.01 (2.42) a	18.72 (2.42) a	19.20 (1.21) a
	Centre	10.73 (2.19) a	10.37 (2.54) a	9.20 (0.90) a
	Tip	7.56 (1.56) a	7.64 (1.31) a	7.40 (1.25) a
B	Base	23.18 (1.80) a	24.22 (1.53) a	22.91 (1.74) a
	Centre	10.86 (1.73) a	11.37 (1.43) a	11.71 (1.24) a
	Tip	9.15 (1.31) a	9.49 (1.25) a	9.58 (0.92) a
A	Base	26.73 (2.32) b	28.39 (2.92) ab	30.17 (2.72) a
	Centre	12.12 (1.44) a	13.46 (2.11) a	13.62 (2.28) a
	Tip	9.81 (1.20) b	10.58 (0.97) b	12.48 (1.97) a

SD, Standard deviation. Values are expressed as mean ± standard deviation (SD). Means followed by the same lowercase letter within a column are not significantly different, while means followed by different lowercase letters indicate significant differences among categories at p < 0.05.

ANOVA results indicated a significant effect of time on cladode thickness at the base for Cambray category (F = 4.87; df = 2, 27; *p* = 0.016). Tukey’s HSD *post-hoc* tests showed that thickness at Time 3 (16.78 ± 1.20 mm) do not have significant differences at Time 1 (16.62 ± 1.3 mm) but was significantly greater than at Time 2 (15.09 ± 1.49 mm), which had the lowest thickness. For Cambray thickness at the center, ANOVA also indicated a significant effect of time (F = 5.79; df = 2, 27; p = 0.008). Tukey’s HSD tests revealed that thickness at Time 3 (9.59 ± 1.21 mm) did not differ significantly from Time 2 (9.38 ± 1.18 mm) but was significantly lower than at Time 1 (11.27 ± 1.63 mm), which had the highest thickness. At the tip of Cambray cladodes, ANOVA indicated a significant effect of time (F = 4.62; df = 2, 27; p = 0.019). Tukey’s HSD tests showed that thickness at Time 3 (7.07 ± 0.95 mm) did not differ significantly from Time 2 (7.45 ± 0.63 mm), while Time 1 (8.16 ± 0.82 mm) did not differ significantly from Time 2.

ANOVA results indicated no significant effect of time on cladode thickness for category C at any position: base (*p* = 0.874), center (*p* = 0.224), and tip (*p* = 0.925). Similarly, for category B, no significant effect of time was observed at the base (p = 0.208), center (*p* = 0.443), or tip (*p* = 0.687).

However, ANOVA results showed a significant effect of time on cladode thickness at the base for category A (F = 4.16; df = 2, 27; *p* = 0.027). Tukey’s HSD *post hoc* tests indicated that thickness at Time 3 (30.17 ± 2.72 mm) did not differ significantly from Time 2 (28.39 ± 2.92 mm), and thickness at Time 1 (26.73 ± 2.32 mm) also did not differ significantly from Time 2.

ANOVA results indicated no significant effect of time on cladode thickness at the center (*p* = 0.198). In contrast, at the tip, ANOVA showed a significant effect of time (F = 8.98; df = 2, 27; *p* = 0.001). Thickness at Time 1 (9.81 ± 1.20 mm) did not differ significantly from Time 2 (10.58 ± 0.97 mm), while thickness at Time 3 (12.48 ± 1.97 mm) was significantly greater than at the other two time points.

### 3.3 Cladode weight

The mean weight of cladodes for each category at each time point is presented in **Table 3**.

**Table 3:** Cladode weight (g) in September 27, September 30, and October 3, 2024.

Category	Time 1 Mean (SD)	Time 2 Mean (SD)	Time 3 Mean (SD)
Cambray	29.55 (10.99) ab	27.01 (10.69) b	38.72 (6.97) a
C	86.25 (29.06) a	81.34 (27.73) a	75.97 (12.47) a
B	202.8 (37.0) a	195.7 (37.0) a	177.1 (45.5) a
A	329.2 (61.2) ab	319.3 (58.3) b	398.4 (82.5) a

SD, Standard deviation. Values are expressed as mean ± standard deviation (SD). Means followed by the same lowercase letter within a column are not significantly different, while means followed by different lowercase letters indicate significant differences among categories at p < 0.05.

ANOVA results indicated a significant effect of Time on cladode weight for Cambray (F = 4.01, df = 2, 27; *p* = 0.015) and A (F = 4.00, df = 2, 27; *p* = 0.030) categories. For Cambray, Tukey’s HSD *post-hoc* tests revealed that the weight at Time 3 (38.72 g) was significantly higher than at Time 2 (27.01 g). For category A, Tukey’s HSD *post-hoc* tests showed that the weight at Time 3 (398.4 g) was significantly higher than at Time 2 (319.3 g). There was no significant effect of time on cladode weight for categories C (*p* = 0.643) and B (*p* = 0.348).

### 3.4 Cladode firmness

The mean texture of cladodes for each category at each time point is presented in **Table 4**.

**Table 4:** Cladode firmness (N) in September 27, September 30, and October 3, 2024.

Category	Time 1 Mean (SD)	Time 2 Mean (SD)	Time 3 Mean (SD)
Cambray	22.25 (2.60) a	22.18 (7.60) a	25.99 (3.31) a
C	16.79 (1.96) b	22.18 (2.51) a	23.96 (2.30) a
B	21.10 (5.74) a	25.31 (2.50) a	25.45 (3.13) a
A	21.59 (2.38) c	27.18 (3.27) b	32.96 (4.49) a

SD, Standard deviation. Values are expressed as mean ± standard deviation (SD). Means followed by the same lowercase letter within a column are not significantly different, while means followed by different lowercase letters indicate significant differences among categories at p < 0.05.

ANOVA results indicated a significant effect of time on cladode texture for categories C (F = 26.95, df = 2, 27; *p* < 0.001), B (F = 3.75, df = 2, 27; *p* = 0.037), and A (F = 26.52, df = 2, 27; *p* < 0.001). Tukey’s HSD *post hoc* tests showed that for category C, texture at Time 3 (23.96 N) did not differ significantly from Time 2 (22.18 N), and both were higher than at Time 1 (16.79 N). For category B, texture at Time 1 (21.10 N) was significantly lower than at Time 2 (25.31 N) and Time 3 (25.45 N). For category A, all time points differed significantly, with textures of 32.56 N at Time 3, 27.18 N at Time 2, and 21.59 N at Time 1. No significant effect of time on cladode texture was observed for the Cambray category (*p* = 0.171).

### 3.5 Cladode color

From **Table 5**, no significant temporal changes in the dominant color tone (hue angle) were observed in any of the four nopal categories during the three measurement points of the observation period. Cambray (*p* = 0.276), C (*p* = 0.210), B (*p* = 0.222) and A (*p* = 0.334). For the chroma, only the Cambray category exhibited a significant change in color saturation over time, showing an increase by Time 3. The other three categories (C, B, and A) maintained relatively consistent color saturation levels during the observation time periods. Only the Cambray category exhibited a significant change in color saturation over time according to Tukey’s HSD *post-hoc* (F = 6.94, df = 2,27 *p* = 0.004), showing an increase by Time 3. The other three categories C (*p* = 0.183), B (*p* = 0.121) and A (*p* = 0.763) maintained rather consistent color saturation levels during the observation periods, these categories did not show significant changes in chroma.

**Table 5:** Hue angle and chroma values in September 27, September 30, and October 3, 2024.

	Time Period		
Category	Time 1 Mean (SD)	Time 2 Mean (SD)	Time 3 Mean (SD)
		Hue angle	
Cambray	154.15 (3.04) a	156.44 (3.46) a	155.95 (3.31) a
C	153.71 (1.92) a	155.75 (3.89) a	155.85 (2.72) a
B	155.35 (5.06) a	152.08 (4.61) a	154.98 (3.71) a
A	151.15 (4.15) a	152.89 (4.23) a	153.79 (3.47) a
		Chroma	
Cambray	24.28 (3.58) b	26.95 (3.69) ab	31.65 (5.81) a
C	26.71 (5.13) a	33.75 (12.53) a	29.46 (4.97) a
B	30.76 (8.34) a	24.32 (2.62) a	28.93 (8.24) a
A	25.00 (6.05) a	27.89 (13.46) a	26.90 (5.78) a

SD, standard deviation. Values are expressed as mean ± standard deviation (SD). Means followed by the same lowercase letter within a column are not significantly different, while means followed by different lowercase letters indicate significant differences among categories at p < 0.05.

**Figure 2:**
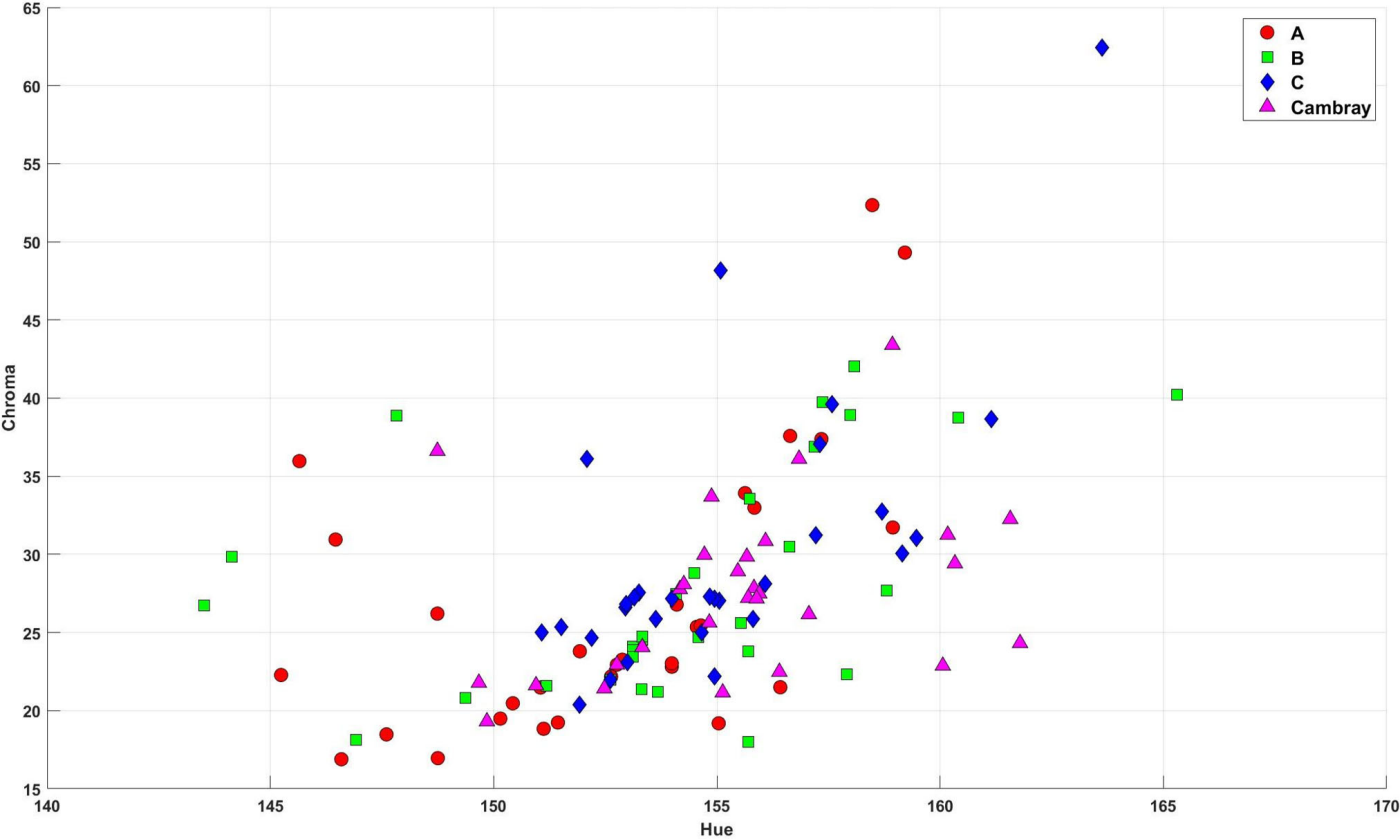
Chroma data of the Cambray, C, B, and A categories of nopal.

The fundamental color of the nopal cladodes did not change much in the short, the intensity or vividness of the color evolved differently in the Cambray category compared to categories C, B, and A (**Figure 2**). These changes may be related to changes in pigment concentration or other factors affecting color expression during this developmental stage.

### 3.6 Chlorophylls and pheophytins

**Figure 3:**
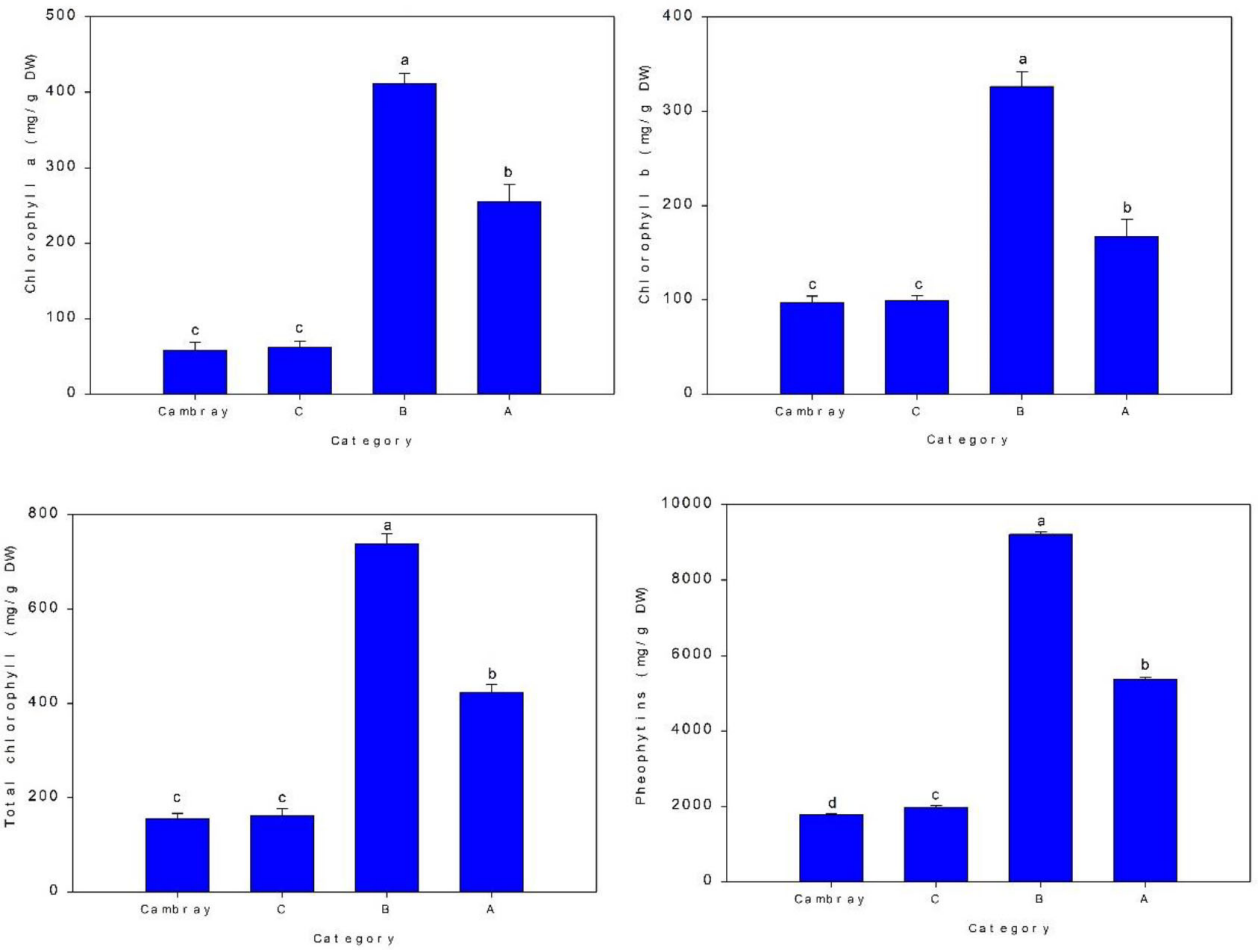
Chlorophyll a, chlorophyll b, total chlorophyll and pheophytin content (mg/g DW) for four nopal categories. DW, Dry weight. Bar is standard deviation. Different letters are significantly differences at *p ≤ 0*.05.

**Figure 3** shows ANOVA results, this indicates that the category B (412 mg/g DW) has the highest level of chlorophyll a, then the category A (256 mg/g DW), and the category Cambray (59 mg/g DW) and category C (63 mg/g DW) with no significative differences have the less content of chlorophyll a. Chlorophyll b showed a similar trend, with category B (327 mg/g DW) having the highest content, then the category A (168 mg/g DW), and the category Cambray (98 mg/g DW) and category C (100 mg/g DW) with no significative differences have the less content of chlorophyll a. For total chlorophylls the category B (739 mg/g DW) has the highest content, then the category A (424 mg/g DW), then the category C (164 mg/g DW) and the category Cambray (157 mg/g DW) has less content. This indicates category B has the highest level of chlorophyll a, b and total chlorophylls. Regarding pheophytins, the highest content was category B (9229.2 mg/g DW), then category A (5391.6 mg/g DW), followed by C (1983.0 mg/g DW) and the last was Cambray (1798.6 mg/g DW).

### 3.7 Ascorbic acid and citric acid

**Figure 4:**
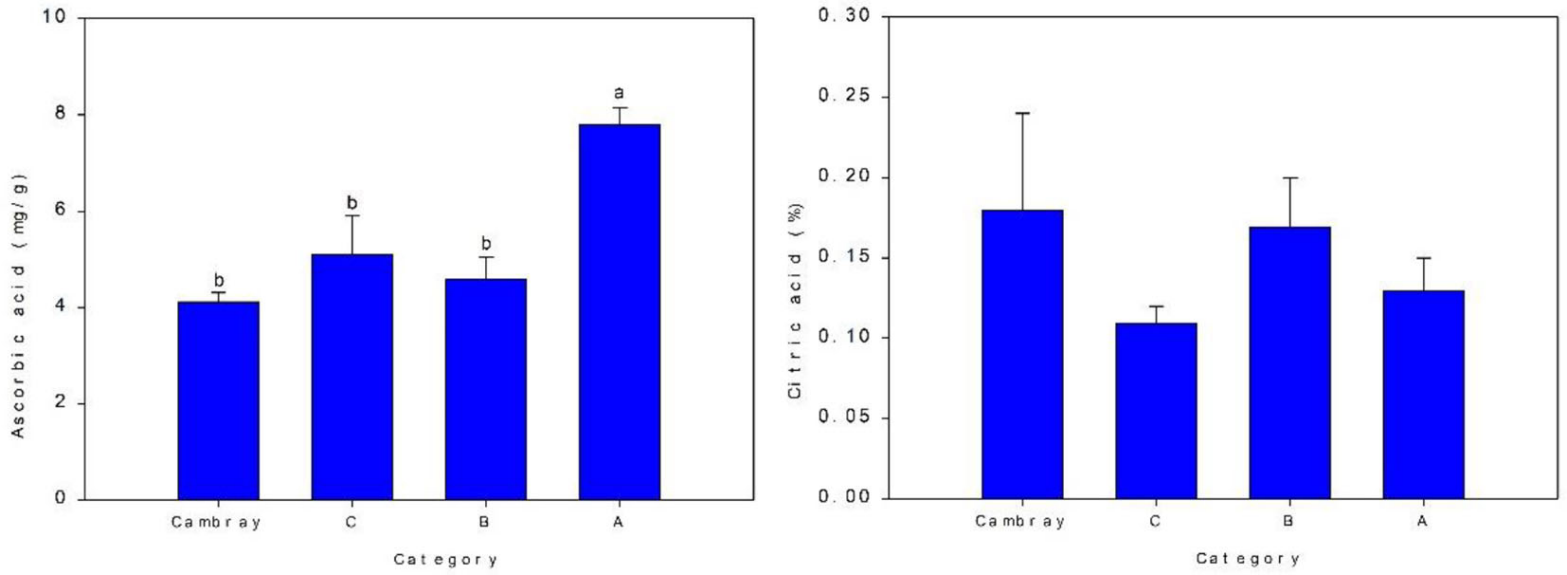
Ascorbic acid (vitamin C) (mg/g) and titratable acidity (%) for four nopal categories. Bar is standard deviation. Different letters are significantly differences at *p ≤* 0.05.

**Figure 4** presents the concentrations of ascorbic acid (vitamin C) and titratable acidity (expressed as % citric acid) across the different size categories (Cambray, C, B, and A). A clear trend was observed in ascorbic acid content, with category A exhibiting the highest concentration (7.81 mg/g), which was significantly greater than the other groups (*p* < 0.05). This suggests that larger cladodes in category A may accumulate more vitamin C, potentially enhancing their nutritional value.

In contrast, titratable acidity, expressed as a percentage of citric acid, did not differ significantly among the size groups. Values ranged narrowly from 0.11% to 0.18%, indicating that cladode size does not markedly influence acidity levels in these samples.

The elevated vitamin C content in the largest size category may be related to metabolic changes during growth or maturity, which could influence antioxidant capacity and taste perception. However, the consistent acidity across size categories suggests that citric acid concentration is maintained regardless of cladode size, possibly reflecting its role in fundamental cellular processes rather than size-dependent metabolic shifts.

These results highlight the importance of cladode size in evaluating nutritional properties, particularly for applications where vitamin C content is a key quality parameter.

### 3.8 Antioxidant capacity

**Figure 5:**
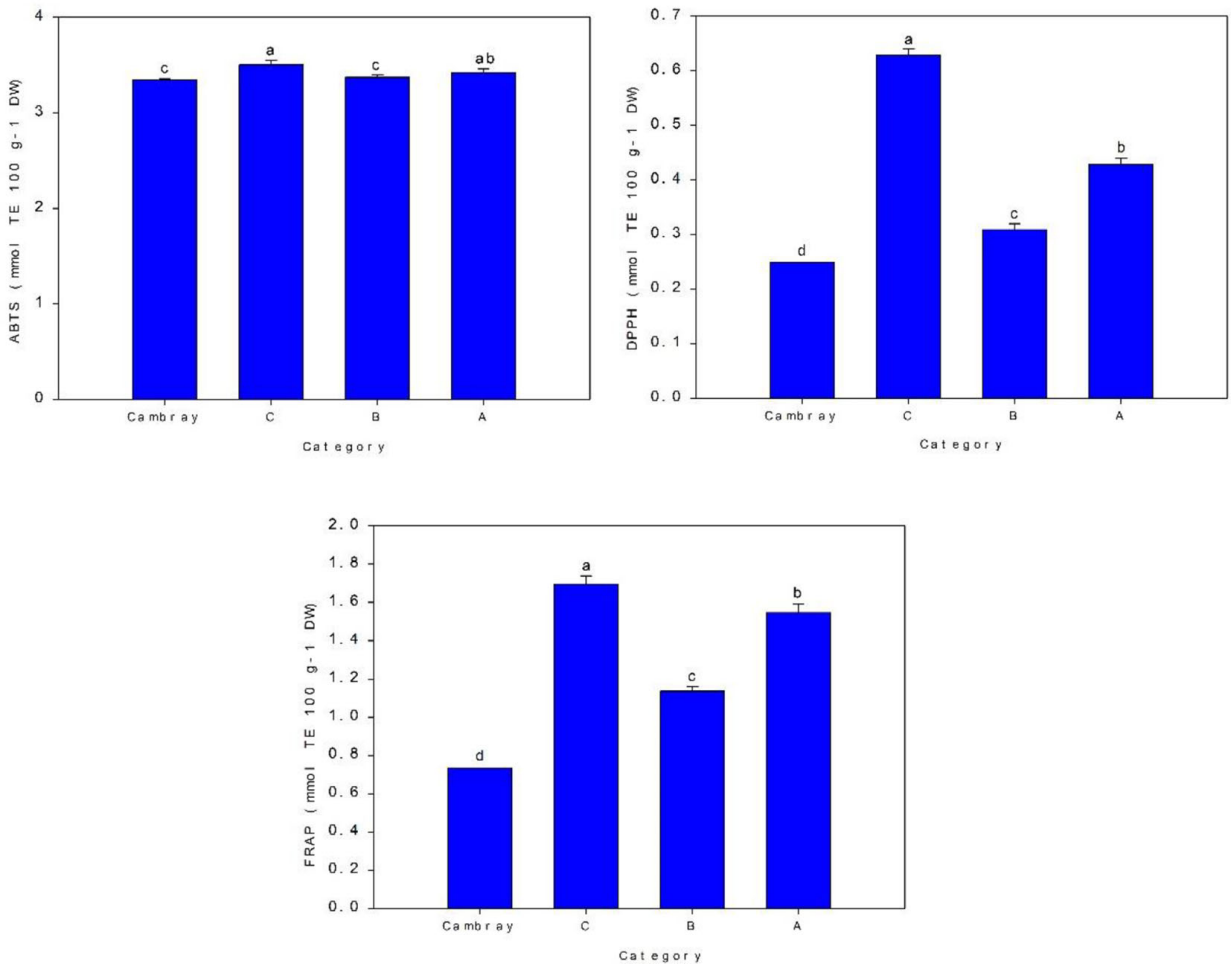
Antioxidant activity (mmol TE 100 g^-1^ DW) for four nopal categories. TE, Trolox Equivalent; DW, Dry weight. Bar is standard deviation. Different letters are significantly differences at *p ≤* 0.05.

Regarding the antioxidant capacity of the cladode, **Figure 5** shows that the analysis of variance results indicated a significant difference between categories. Category C had the highest antioxidant activity ABTS (3.51 mmol TE 100 g^-1^ DW), while the lowest value was for Cambray (3.35 mmol TE 100 g^-1^ DW) and B (3.51 mmol TE 100 g^-1^ DW). For DPPH, it records the highest value (0.63 mmol TE 100 g^-1^ DW), followed by A (0.43 mmol TE 100 g^-1^ DW), B (0.31 mmol TE 100 g^-1^ DW) and Cambray (0.25 mmol TE 100 g^-1^ DW). The FRAP showed a similar trend, with C (1.70 mmol TE 100 g^-1^ DW) showing the highest value, indicating the greatest reducing power, and Cambray (0.74 mmol TE 100 g^-1^ DW) showing the weakest performance in this assay as well. Results indicated a significant difference in cladode antioxidant capacity for categories.

### 3.9 Total phenolics content and flavonoids

**Figure 6:**
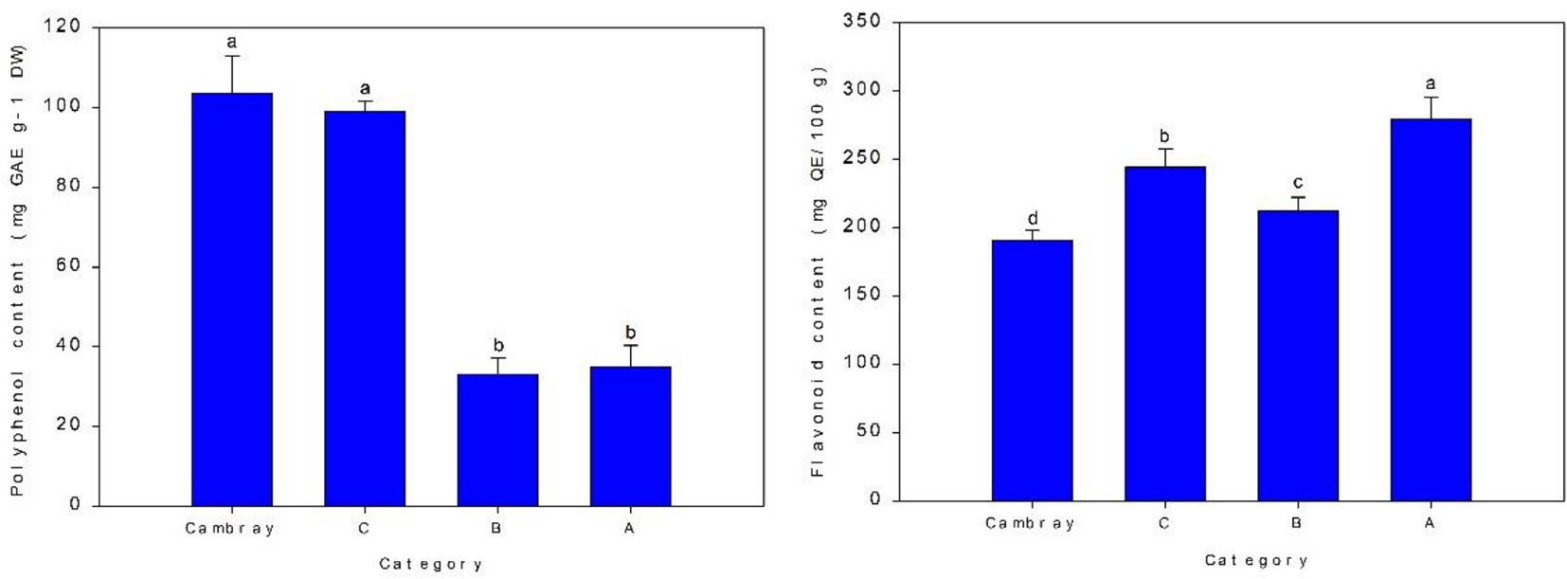
Total polyphenol content (mg GAE g^-1^ DW) and flavonoid content (mg QE/100 g) for four nopal categories. GAE, Gallic acid equivalent; QE, Quercetin equivalent; DW, Dry weight. Bar is standard deviation. Different letters are significantly differences at *p ≤* 0.05.

**Figure 6** presents the results of the analysis of variance for total polyphenol and flavonoid contents. Cambray (103.8 mg GAE g^−1^ DW) and category C (99.2 mg GAE g^−1^ DW) exhibited the highest total polyphenol content, while categories B (33.4 mg GAE g^−1^ DW) and A (10.39 mg GAE g^−1^ DW) showed the lowest, with no significant differences observed between them. In contrast, category A had the highest flavonoid content (280.5 mg QE/100 g DW), followed by C (244.8 mg QE/100 g DW), B (212.8 mg QE/100 g DW), and Cambray (191.1 mg QE/100 g DW).

## 4 Discussion

Nopal is characterized by a short postharvest period (Liguori and Inglese, 2013). Currently, strategies such as the application of edible coatings or storage at low temperatures have been explored to extend shelf life. Previous studies have shown that the physicochemical properties of mucilage are influenced by cladode size (Costa de Sousa et al., 2022), suggesting that size may also affect postharvest longevity. After harvest, metabolic processes continue, with transpiration leading to loss of cellular turgor, softening, diminished shine, and other quality changes (Trindade et al., 2023). These processes justify the evaluation of the parameters considered in this study.

Color is a key indicator of visual quality for consumers, with bright green being associated with high-quality products. However, this color can change during storage due to factors such as light and temperature (Andreu-Coll et al., 2021). In the present study, hue angle values ranged from 151° to 157° at room temperature, compared to 178°–180° reported by Meraz-Maldonado et al. (2012) under refrigeration. This difference highlights the significant effect of temperature, which is a major factor influencing the rate of deterioration in fruits (Hossain et al., 2024).

Cladode color is also influenced by the presence of pigments such as chlorophyll. Category B exhibited the highest chlorophyll content, which correlates with better perceived quality. However, thermal processing and acidification can convert chlorophyll into pheophytins, resulting in an olive-green coloration of the tissue (Reyes-Escogido et al., 2025). Category B also had the highest pheophytin levels, indicating a possible interaction between processing conditions and pigment stability.

A study by dos Santos et al. (2024) evaluated *Opuntia ficus-indica* cladodes and found they could be stored for up to 56 days without reductions in crude protein or neutral detergent fiber. However, soluble carbohydrate content in water decreased with longer storage times, potentially compromising nutritional value. This may partly explain the observed decrease in firmness in categories C, A, and B, as firmness loss is primarily caused by cell desiccation during storage, leading to reduced turgor pressure (Ali et al., 2014).

Titratable acidity reflects the total concentration of acids in foods, influencing flavor, color, preservation, and other quality attributes, and is commonly used as an indicator of ripeness (Sadler and Murphy, 2010). Consistent with this study’s findings, Andreu et al. (2017) reported no significant differences in titratable acidity among cladode sizes, with values of 3.49 and 2.80 g/L for young and mature cladodes, respectively. Regarding citric acid content, in contrast to Betancourt-Domínguez et al. (2006), who reported 0.30%, 0.33%, and 0.34% for small, medium, and large white-spined nopal, respectively, the values obtained here were lower, suggesting varietal or environmental influences.

In addition to visual and physicochemical quality, nutraceutical properties are increasingly important. Ascorbic acid is essential for proper human metabolism, acting as an antioxidant and regulating reactive oxygen species (Gęgotek and Skrzydlewska, 2023). Galvão et al. (2018) reported ascorbic acid content of 2.4 mg/100 g in Atlixco cladodes, whereas the present study found values ranging from 4.13 to 7.81 mg/100 g, indicating a substantial nutritional contribution and potential metabolic significance (Foyer, 2017).

Regarding polyphenols, the Cambray and C categories exhibited the highest content. This aligns with findings by Figueroa-Pérez et al. (2018), who reported that young cladodes generally have higher polyphenol levels than older ones. Polyphenols, as secondary metabolites, are well-known for their beneficial effects on human health, further supporting the functional value of these nopal categories.

## 5 Conclusion

This study evaluated cladodes of *Opuntia ficus-indica* (nopal) in categories Cambray, C, B, and A for 10 days after harvest. Regarding size, chroma, and hue angle, no significant variations were observed among the categories. For thickness, categories C and B did not show significant differences, while Cambray exhibited variation between the base, center, and tip, and category A showed differences between the base and tip. In terms of weight, categories C and B remained unchanged, whereas Cambray and category A presented significant differences. Firmness varied in Cambray and category B, but no differences were detected in categories C and A.

Category B exhibited the highest levels of chlorophyll a, chlorophyll b, total chlorophyll, and pheophytins. Category A showed the highest ascorbic acid and flavonoid contents, while category C had the highest DPPH, FRAP, and total polyphenol levels.

Comparing the categories and considering the post-harvest handling of the nopal, the best performance was observed in the category that maintained stability in size, firmness, thickness, and color, while also exhibiting the highest levels of chlorophyll a, b, total chlorophyll, and pheophytins.

These findings provide new insights into the developmental dynamics and biochemical diversity of nopal cladodes, with important implications for both agricultural management and value-added industrial applications. Even within a very short evaluation period, differences were observed in multiple parameters, enabling the identification of new evaluation criteria and time points for future research.

## Data Availability

The raw data supporting the conclusions of this article will be made available by the authors, without undue reservation.
